# Neighborhood and Psychosocial Predictors of Cognitive Function Among Latinos in the United States

**DOI:** 10.1093/geroni/igaf122.1384

**Published:** 2025-12-31

**Authors:** Angela Gutierrez, Alejandra Marroig, Courtney Thomas Tobin, María Aranda, David Camacho, Roland Thorpe, Barış Sevi, Graciela Muniz-Terrera

**Affiliations:** Western University of Health Sciences, Pomona, California, United States; Universidad de la Republica Uruguay, Montevideo, Montevideo, Uruguay; University of California, Los Angeles Fielding School of Public Health, Los Angeles, California, United States; University of Southern California, Los Angeles, California, United States; University of Illinois Chicago, Chicago, Illinois, United States; Johns Hopkins University, Baltimore, Maryland, United States; MEF University, Maslak, Istanbul, Turkey; Ohio University, Athens, Ohio, United States

## Abstract

Prior research highlights the independent roles of neighborhood and psychosocial risk and protective factors for accelerated cognitive decline. However, the combined role of risk and protective neighborhood and psychosocial factors for cognitive aging among middle-aged and older Latinos in the U.S. remains unclear. Utilizing data from Latinos aged 50 and older (n = 2,321) in the Health and Retirement Study (2006 - 2016), this study aimed to examine the role of neighborhood (perceived physical disorder, neighborhood social cohesion) and psychosocial (loneliness) factors in shaping baseline cognitive function and change in cognitive function. Linear mixed models were used to estimate baseline and rate of change in cognitive function, adjusting for sociodemographic factors. Neighborhood social cohesion was positively associated with baseline cognitive function (p < 0.001), while perceived neighborhood social disorder and loneliness were both negatively associated with baseline cognitive function (p < 0.001). However, none of these factors were significantly associated with cognitive trajectories over time. These findings highlight the role of psychosocial and neighborhood risk and protective factors in baseline cognitive function among Latino adults. To promote healthy aging, health promotion efforts should consider implementing culturally relevant strategies to strengthen neighborhood social cohesion and reduce loneliness.

